# Somatosensory profiles of patients with chronic myogenic temporomandibular disorders in relation to their painDETECT score

**DOI:** 10.1186/s12903-018-0601-8

**Published:** 2018-08-09

**Authors:** C. Welte-Jzyk, D. B. Pfau, A. Hartmann, M. Daubländer

**Affiliations:** 1 Department of Oral and Maxillofacial Surgery, University Medical Centre of the Johannes Gutenberg University of Mainz, Mainz, Germany; 2 Mannheim Institute of Public Health (MIPH), Social and Preventive Medicine, University of Heidelberg, Heidelberg, Germany; 3 Department of Neurophysiology, Centre of Biomedicine and Medical Technology Mannheim (CBTM), University of Heidelberg, Heidelberg, Germany; 4 Private Practice Dr. Seiler and colleagues, Filderstadt, Germany

**Keywords:** Temporomandibular disorder (TMD), Quantitative sensory testing (QST), PainDETECT questionnaire, Stress-induced hyperalgesia

## Abstract

**Background:**

The purpose of this study was to characterize patients with chronic temporomandibular disorders (TMD) in terms of existing hyperalgesia against cold, heat and pressure.

**Methods:**

The extent of hyperalgesia for pressure and thermal sensation in TMD patients was determined by the use of the painDETECT questionnaire ("Is cold or heat in this area occasionally painful?” “Does slight pressure in this area, e.g., with a finger, trigger pain?") and experimental somatosensory testing against thermal and pressure stimuli (Quantitative Sensory Testing; QST). In addition, we explored psychological comorbidity among the chronic TMD patients (hospital anxiety and depression scale, HADS-D and coping strategies questionnaire, CSQ).

**Results:**

Nineteen patients with chronic TMD and 38 healthy subjects participated in the study. *N* = 12 patients had a painDETECT score ≤ 12, *n* = 3 patients had a painDETECT score of 13–18 and *n* = 4 patients had a painDETECT score ≥ 19. TMD patients with painDETECT scores ≥19 had moderately, strong or very strong enhancement of thermal and pressure pain perception, whereas patients with painDETECT scores 13–18 and ≤ 12 responded these questions with “never”, “hardly noticed” or “slightly painful” (*p* < 0.05–0.01). With increasing painDETECT scores we found increased hyperalgesia for pressure (*p* < 0.01) and thermal stimuli (*p* < 0.05) in QST. The patients with a painDETECT score ≥ 19 showed increased signs of anxiety (*p* < 0.05), depression (*p* < 0.01), praying and hoping (*p* < 0.05).

**Conclusion:**

The present study has shown that the PainDETECT questionnaire can be a helpful additional diagnostic tool. Together with QST, the PainDETECT questionnaire detected hyperalgesia for pressure and thermal sensation. Therefore the PainDETECT questionnaire is helpful to decide which TMD patients should undergo QST.

## Background

Temporomandibular disorder (TMD) is an umbrella term for various pathological conditions characterized by pain and/or dysfunction of the masticatory muscles and/or the temporomandibular joint [1–4]. TMD is the major cause of non-dental chronic facial pain [1, 2, 5] with an estimated prevalence of 3 to 5% in the general population [5]. Since the aetiology of TMD is multidimensional, including physiological, structural, postural, psychological and genetic factors [4, 6–8], a complex diagnostic approach for TMD is required in clinical diagnostics, treatment and research [9, 10]. Notably, the population with chronic myogenic TMD is very heterogeneous [11, 12]. Apart from “sensitive” and “insensitive” myogenic TMD pain patients, as found in a previous study [11], there were also TMD patients who respond or not to standard conservative therapy alone or in combination with cognitive behavioral therapy [12]. The characteristics of “non-responding” patients did not differ on demographics or temporomandibular joint pathology, but showed higher psychological comorbidity such as poorer coping strategies and higher levels of catastrophizing [12]. At the same time, psychological distress such as depression, anxiety, and somatization contribute to the progression of TMD [8, 13–15], whereby effective pain management is complicated due to the interaction of all these factors [16, 17]. Repeated episodes of pain and continuous nociceptive input may shift the balance of central modulation, contributing to sustained chronic pain [18]. For these patients, spontaneous pain most often is not only present in the area of the trigeminal nerve, but throughout the whole body. This condition is called hyperalgesia and can be interpreted as central sensitization with insufficient endogenous descending inhibition [11, 18] and is induced by persistent psychosocial stress and increased mental vulnerability [19, 20]. Stress and anxiety exert modulatory influences on pain depending on the nature, duration and intensity of the stressor and developmental influences on the maturation of the stress and pain system [21]. In this context there is a bidirectional relationship between psychological comorbidity and spontaneous myogenic pain. To reveal such stress-induced hyperalgesia, a comprehensive analysis of the medical history and a careful clinical examination is required in the diagnostic process of chronic TMD patients.

The purpose of this study was to characterize patients with chronic myogenic TMD in terms of existing hyperalgesia for pressure and thermal sensation.

With the painDETECT questionnaire it is possible to ask for the subjective rating of the patient of the extent of hyperalgesia to cold or heat and pressure. Those modalities can also be experimentally assessed with the Quantitative Sensory Testing (QST) protocol.

Furthermore, we looked for psychological comorbidity among the TMD patients as trigger factors of hyperalgesia.

## Methods

The study followed the 1964 Declaration of Helsinki on medical protocol and ethics. Ethical approval was obtained from the local ethical committee (ethics committee of Rhineland-Palatinate, no.837.067.09 (6572)). The study was designed as a prospective clinical monocenter study at the Department of Oral and Maxillofacial Surgery, University Medical Centre of the Johannes Gutenberg University of Mainz, Germany. Written consent was obtained from all patients and volunteers prior to the study.

### Patients

Nineteen patients with myogenic temporomandibular disorders (TMD) were selected from the pain outpatient clinic of the Department of Oral and Maxillofacial Surgery, University Medical Centre of the Johannes Gutenberg University of Mainz, Germany as diagnosed by one investigator (MD) using the Research Diagnostic Criteria for TMD [22]. Inclusion criteria were chronic uni- or bilateral myogenic pain (duration ≥6 months). Only patients with bilateral intact or prosthetically supported occlusion were included. Diseases that interfere with pain perception or cause pain in other body regions were an exclusion criterion. Also excluded were patients consuming antidepressant or anticonvulsive drugs, as well as those having taken drugs influencing pain perception (analgesics) within the last 24 h.

TMD patients underwent careful clinical examination as stated below to reveal abnormalities in facial sensibility, muscle and temporomandibular joint sensitivity to palpation, mandibular movement and auscultation of the joint. Additionally, comprehensive medical history as well as pain history was documented on paper-based charts. QST was performed and the psychological comorbidity determined.

We first analysed the patients as a collective group (TMD all) and then divided them into 3 groups depending on the patient’s painDETECT score (≤ 12, *n* = 12; 13–18, *n* = 3; ≥ 19, *n* = 4).

### Control group (healthy subjects)

Patients were compared to healthy subjects who had had no temporomandibular disorder complaints during the last 6 months. Healthy subjects were recruited by an announcement in the department of Oral and Maxillofacial Surgery, University Medical Centre of the Johannes Gutenberg University of Mainz, Germany. Patients were compared related to clinical investigation, QST values and psychological comorbidity. Included were healthy subjects not having taken drugs influencing pain perception (analgesics) within the last 24 h.

### Clinical investigation

Patients and healthy subjects were examined by one investigator (MD) according to dental and clinical factors to reveal abnormalities in countenance, mandibular movement, auscultation of the jaw, tension of the facial muscles, and facial sensitivity. Examination was carried out as described in Pfau et al. 2009 [11].

For intraoral dental examination, the dentition and static contacts were noted as well as signs of oral habits. We determined the total number of missing teeth, the number of missing teeth having been replaced by removable dentures or bridges, and the numbers of crowned or filled teeth. Distances of overbite, overjet and interocclusal distances were measured. For extraoral examination, we tested the functions of muscles, nerves and the movements of the temporomandibular joint. We investigated the function of the facial nerve and the sensitivity to pressure over trigeminal foramina. Signs of underlying myogenic orofacial hyperactivity were documented after checking the mimic muscles, masticatory and neck muscles. Temporalis, masseter muscle, sternocleidomastoid muscle, muscles of the cervical spine, trapezius muscle and suprahyoidal muscles were palpated with the fingertips of the index and the third finger, using the non-dominant hand of the investigator to fix the head e.g. the mandible. For palpation of the extra oral muscles we used an approximate pressure of ~ 10 N, for intraoral muscle and joint palpation an approximate pressure of ~ 5 N. We checked if a single active mandibular movement (forward movements, laterotrusion and mouth opening) or an assisted backward movement was painful. The diagnostic findings (unpleasant, painful, trigger point) during palpation of temporalis, medial and lateral pterygoid and masseter muscle were also documented.

### Pain history and current pain

The medical records and pain history of the patients were taken in a written form and completed verbally. Patients were asked to answer different questionnaires on pain, including questions on pain intensity, pain duration, and pain localisation. We used the Berne pain questionnaire (BPQ, paper-based chart) consisting of 20 groups of sensory, affective, and evaluative items to describe the quality and intensity of the pain [23] and the painDETECT paper-based form [24]. The painDETECT questionnaire was developed and validated for patients with neuropathic pain and is increasingly applied to patients with back pain [25, 26]. It consists of questions concerning estimation of pain intensity, pain duration, pain patterns (persistent pain and or pain attacks) and pain quality (burning, tingling or prickling sensation, numbness, and temperature and pressure hyperalgesia). We used the painDETECT questionnaire as it contains questions concerning hyperalgesia against cold or heat and pressure (“Is cold or heat in this area occasionally painful?” “Does slight pressure in this area, e.g., with a finger, trigger pain?”) Patients were divided into 3 groups according to their PainDETECT score. In the group “≤ 12” a neuropathic component is unlikely, in the group “≥ 19” a neuropathic component is likely, and in the group “13–18”, it is uncertain whether a neuropathic component exists. The painDETECT questionnaire demonstrated satisfactory reliability [27], showing accurate test-retest stability as a prerequisite for use in repeated measurements [25].

### Psychological testing

In order to assess psychological comorbidity, patients and healthy subjects were asked questions relating to coping strategies (CSQ, full paper-based version), and questions on anxiety and depression disorders (HADS-D; hospital anxiety and depression scale, German version, full paper-based charts).

### Quantitative sensory testing (QST)

Changes of thermal and mechanical detection and pain thresholds were examined using the Quantitative Sensory Testing protocol according to the DFNS (Deutscher Forschungsverbund Neuropathischer Schmerz), described in detail by Rolke et al. [28, 29] and Hartmann et al. [30].

The QST protocol consists of the following parameters: CDT (cold detection threshold); WDT (warm detection threshold); TSL (thermal sensory limen); PHS (paradoxical heat sensation); CPT (cold pain threshold); HPT (heat pain threshold); MDT (mechanical detection threshold); MPT (mechanical pain threshold); MPS (mechanical pain sensitivity); DMA (dynamic mechanical allodynia); WUR (wind up ratio); VDT (vibration detection threshold) and PPT (pressure pain threshold).

All patients and healthy subjects underwent the same QST protocol. They were tested on the left and on the right masseter muscles by one trained examiner within one experimental session, which took roughly 60 min.

In a first step, the patients were examined as a whole group and then divided into 3 groups according to their painDETECT score (≤ 12; 13–18; ≥ 19).

### Z-transformation of QST data

To compare patients’ QST data profiles with the age and gender-matched healthy subjects, reference data of healthy subjects were used to normalize test results of patients by calculating the z-transform: Z = (value (patient) – mean (healthy subjects))/ standard deviation (healthy subjects). This procedure results in a QST profile where all parameters are presented as standard normal distributions (zero mean, unit variance). Z-values above “0” indicate a gain of function when the single patient is more sensitive to the tested stimuli compared with controls (hyperalgesia, allodynia, hyperpathia), while Z-scores below “0” indicate a loss of function referring to a lower sensitivity of the patient (small and large fibre functions). A Z-score of zero represents a value corresponding to the group mean of the healthy control subjects.

### Statistical analysis

#### QST data analysis

Data evaluation was performed according to the standardized protocol of the German Research Network on Neuropathic Pain [28, 29]. All data were normally distributed in log-space and were transformed logarithmically before statistical analysis, with the exception of the CPT, HPT and VDT number, which were normally distributed as raw data. All statistical calculations were performed using SPSS software (IBM SPSS Statistics 23) or Excel for Windows (Microsoft Excel 2010). All data are presented as mean ± standard error of the mean (SEM). Differences of QST data between patient groups and control group (healthy subjects) were determined using an unpaired t-test, considering the Levene’s test for equality of variances for comparison of TMD all with healthy subjects or analysis of variance (ANOVA) with LSD post hoc corrections of multiple comparisons for comparison of the different painDETECT subgroups with healthy subjects. The significance level was set at *p* < 0.05.

### Data of clinical examination and questionnaires

All statistical calculations were performed using SPSS software (IBM SPSS Statistics 23) or Excel for Windows (Microsoft Excel 2010). All data are presented as mean ± standard deviation (SD). Differences between patient groups and control group (healthy subjects) were determined using an unpaired t-test, considering the Levene’s test for equality of variances for comparison of TMD all with healthy subjects or analysis of variance (ANOVA) with LSD post hoc corrections of multiple comparisons for comparison of the different painDETECT subgroups with healthy subjects. The level of significance was set at *p* < 0.05.

## Results

### Patients

Nineteen patients with myogenic temporomandibular disorders (TMD) were included as diagnosed by one investigator (MD) using the Research Diagnostic Criteria for TMD [22]. The patients (13 women, 6 men) had a mean age of 52.2 ± 14.9 years. Fifteen patients had unilateral symptoms of temporomandibular disorder, 7 patients on the left side, 8 patients on the right side and 4 patients had bilateral findings.

### Control group (healthy subjects)

Thirty-eight healthy subjects (33 women, 5 men) with a mean age of 46.7 ± 13.3 years were included.

### Clinical findings

#### TMD patients (TMD all)

**Fig. 1:**
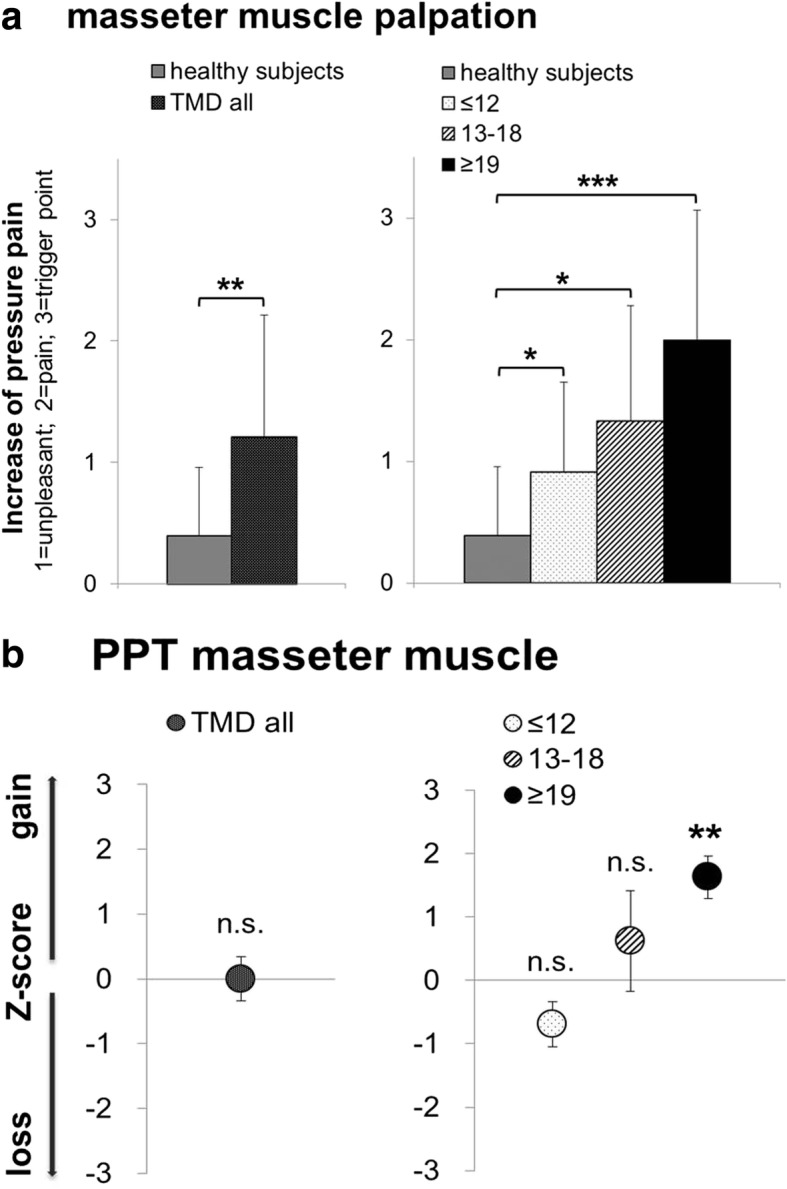
**a** Increase of pressure pain from unpleasant over painful to trigger point on masseter muscle palpation in patients with TMD (TMD all, *n* = 19; TMD subgroups concerning their painDETECT score (≤ 12, *n* = 12; 13–18, *n* = 3; ≥ 19, *n* = 4); mean ± SD (masseter left + right); **b** QST-pressure pain threshold (PPT) presented as z-score values (mean value of patients (masseter left + right) - mean controls (masseter left + right)/SD controls). A z-score of 0 means the score is the same as for the mean of healthy subjects. It can also be negative or positive indicating a loss or gain of function; *p*-value for TMD all as results of unpaired t-test as related to healthy subjects considering the Levene’s test for equality of variances; p-value according to the painDETECT scores as results of analysis of variance (ANOVA) with LSD post hoc correction of multiple comparison; (n.s. = not significant, * = *p* < 0.05, ** = *p* < 0.01, *** = *p* < 0.001)

Regarding the patients with chronic TMD in its entirety (TMD all) we found no changes in the function of mimic muscle. TMD patients (TMD all) showed increased sensitivity towards palpation of the trigeminal foramina, but only palpation of the left infraorbital foramen was significantly more painful (*p* < 0.05). TMD patients (TMD all) showed increased sensitivity towards palpation of the masticatory muscles. In particular, the pain values for the temporalis (left *p* < 0.05; right *p* < 0.001), the masseter (left *p* < 0.05; right *p* < 001, masseter left + right *p* < 0.01, Fig. 1a) and the sternocleidomastoid muscle (left *p* < 0.05; right *p* < 0.01) were increased. It should be noted that, with regards to the palpation of the masticatory muscles, particularly the masseter, that also the healthy subjects (*n* = 12, 31%) showed pain upon palpation. TMD patients (TMD all) showed increased sensitivity to palpation of the temporomandibular joint (*p* < 0.01), particularly during dorsal palpation. Furthermore, TMD patients (TMD all) showed pain when opening the mouth (*p* < 0.01) and during laterotrusion (*p* < 0.01).

#### TMD subgroups as a function of painDETECT scores

Examining the patients with chronic TMD according to their painDETECT scores, we found patients within the “painDETECT ≥ 19” subgroup showing notably increased sensitivity towards palpation of the temporalis (left *p* < 0.001; right *p* < 0.001), the sternocleidomastoid muscle (left *p* < 0.05; right *p* < 0.01) and the masseter muscle (left *p* < 0.01; right *p* < 0.001; masseter left + right *p* < 0.001, Fig. 1a). Furthermore, sensitivity towards palpation of the temporomandibular joint was enhanced in the “painDETECT ≥ 19” subgroup (lateral *p* < 0.05 and dorsal *p* < 0.01). In addition, opening the mouth was painful (*p* < 0.05) and forward movements (protrusion; *p* < 0.001) in the TMD subgroup with painDETECT scores ≥ 19.

### QST findings

#### TMD patients (TMD all)

The significant differences in masseter pressure pain sensitivity found in clinical examination for the TMD patients in its entirety (TMD all), compared to the healthy subjects (Fig. 1a), *p* < 0.01), could not be confirmed in QST pressure pain thresholds (PPT) (Fig. 1b, Table 1). Furthermore, there were no differences for all other QST parameter, except for VDT, whereby the TMD patients showed a higher sensitivity than the healthy subjects (Table 1, *p* < 0.05).

**Table 1:** QST data of the masseter of patients with TMD (TMD all and according to their painDETECT scores (≤ 12., *n* = 12; 13–18, *n* = 3; ≥ 19, *n* = 4)); CPT, HPT, VDT (Data in original unit as mean ± SEM); CDT, WDT, TSL, MDT, MPT, MPS, WUR, PPT (retransformed log data (mean log ± SEM))

masseter	Healthy subjects	TMD all		TMD - painDETECT					
	(*n* = 38)	(*n* = 19)		≤ 12 (*n* = 12)		13–18 (*n* = 3)		≥ 19 (*n* = 4)	
	QST data raw mean ± SEM) or log retransformed (mean_log_ ± SEM)	QST data raw mean ± SEM or log retransformed (mean_log_ ± SEM)	*p*-value	QST data raw mean ± SEM or log retransformed (mean_log_ ± SEM)	*p*-value	QST data raw mean ± SEM or log retransformed (mean_log_ ± SEM)	*p*-value	QST data raw mean ± SEM or log retransformed (mean_log_ ± SEM)	*p*-value
CDT (Δ°C)	1.2 (0.078 ± 0.024)	1.39 (0.142 ± 0.0579)	0.327	1.62 (0.209 ± 0.082)	0.041*	1.08 (0.033 ± 0.058)	0.691	1.05 (0.022 ± 0.031)	0.565
WDT (Δ°C)	1.84 (0.265 ± 0.029)	2.23 (0.348 ± 0.056)	0.164	2.63 (0.420 ± 0.079)	0.027*	1.82 (0.259 ± 0.041)	0.959	1.58 (0.199 ± 0.067)	0.541
TSL (°C)	2.78 (0.443 ± 0.031)	3.42 (0.534 ± 0.069)	0.181	4.22 (0.625 ± 0.093)	0.02*	3.07 (0.487 ± 0.059)	0.752	1.97 (0.294 ± 0.067)	0.218
CPT(°C)	16.54 ± 1.35	14.59 ± 2.44	0.500	10.53 ± 2.63	0.043*	15.23 ± 6.1	0.804	26.3 ± 2.15	0.038*
HPT (°C)	42.99 ± 0.74	43.62 ± 1.26	0.655	45.92 ± 1.38	0.062	43.09 ± 2.68	0.972	37.14 ± 0.56	0.02*
MDT (mN)	0.42 (−0.375 ± 0.053)	0.34 (− 0.469 ± 0.058)	0.290	0.31 (− 0.507 ± 0.065)	0.209	0.56 (− 0.253 ± 0.189)	0.509	0.3 (−0.518 ± 0.079)	0.388
MPT (mN)	41.44 (1.617 ± 0.067)	51.33 (1.710 ± 0.108)	0.458	39.94 (1.601 ± 0.122)	0.909	204.46 (2.311 ± 0.130)	0.009**	38.67 (1.587 ± 0.20)	0.893
MPS (0–100)	0.34 (− 0.471 ± 0.099)	0.36 (− 0.447 ± 0.136)	0.883	0.36 (−0.440 ± 0.114)	0.851	0.08 (− 1.119 ± 0.114)	0.073	1.08 (0.034 ± 0.387)	0.116
WUR (0–100)	3.96 (0.598 ± 0.061)	3.35 (0.526 ± 0.063)	0.565	3.25 (0.512 ± 0.080)	0.539	not evaluable		3.73 (0.572 ± 0.133)	0.911
VDT (x/8)	6.02 ± 0.11	6.56 ± 0.2	0.014*	6.86 ± 0.25	0.001**	5.83 ± 0.21	0.684	6.21 ± 0.36	0.622
PPT (kPa)	191 (2.281 ± 0.025)	191 (2.281 ± 0.052)	0.920	244 (2.387 ± 0.054)	0.066	154 (2.186 ± 0.120)	0.305	108 (2.034 ± 0.051)	0.004**

*CDT* Cold detection treshold, *WDT* Warm detection threshold, *TSL* Thermal sensory lumen, *CPT* Cold pain threshold, *HPT* Heat pain threshold, *MDT* Mechanical detection threshold, *MPT* Mechanical pain threshold, *MPS* Mechanical pain sensitivity, *WUR* Wind up ratio, *VDT* Vibration detection threshold, *PPT* Pressure pain threshold, *p*-value for TMD all as results of unpaired t-test as related to healthy subjects considering the Levene’s test for equality of variances; *p*-value according to the painDETECT scores as results of analysis of variance (ANOVA) with LSD post hoc correction of multiple comparison; (*p < 0.05; **p < 0.01; ***p < 0.001)

#### TMD subgroups as a function of painDETECT scores

**Fig. 2:**
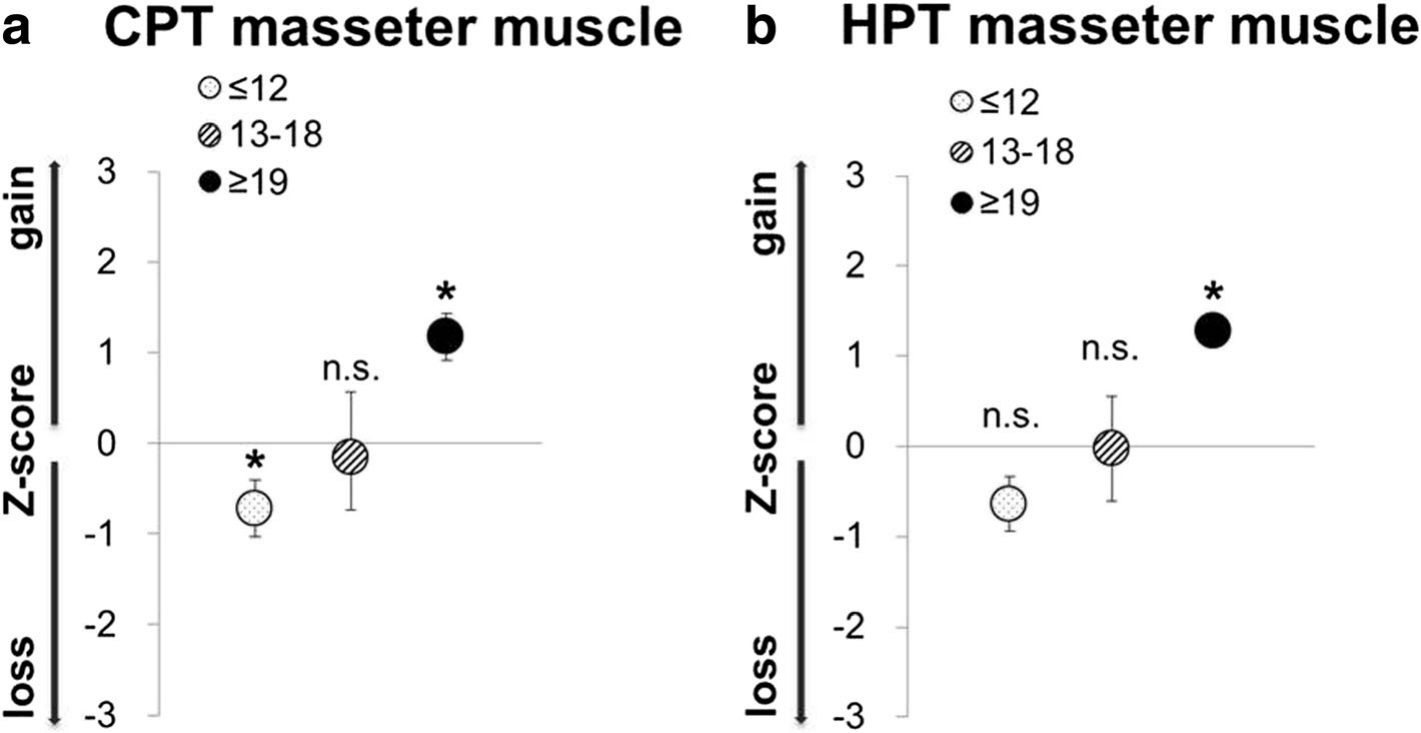
Thermal hyperalgesia in patients with myogenic TMD concerning their painDETECT score (≤ 12, n = 12; 13–18, n = 3; ≥ 19, n = 4) **a** QST-Cold pain threshold (CPT) and **b** QST-Heat pain threshold (HPT) presented as z-score values (mean value of patients-mean controls/SD controls). A z-score of 0 means the score is the same as for the mean of healthy subjects. It can also be negative or positive indicating loss or gain of function; Significance as results of analysis of variance (ANOVA) with LSD post hoc correction of multiple comparison; (n.s. = not significant, * = *p* < 0.05, ** = *p* < 0.01, *** = *p* < 0.001)

When analysing the results of patients with chronic TMD according to their painDETECT scores, we found similar results for palpation of the masseter muscle (Fig. 1a), whereas totally different results emerged for the QST PPT values. In QST we found the TMD patients with a painDETECT score ≥ 19 showing significant higher sensitivity to pressure (PPT) compared to healthy subjects (Fig. 1b, Table 1, *p* < 0.01). Patients with a painDETECT score ≥ 19 were also significantly more sensitive to painful cold (CPT) and painful heat (HPT) compared to healthy subjects (Fig. 2, Table 1, *p* < 0.05; *p* < 0.05), whereas patients showing painDETECT scores ≤ 12 were less sensitive to cold (CPT) than healthy subjects (Fig. 2, Table 1, *p* < 0.05). The results underline the finding that TMD pain patients appear to be a heterogeneous group.

### Findings of the painDETECT questionnaire

**Fig. 3:**
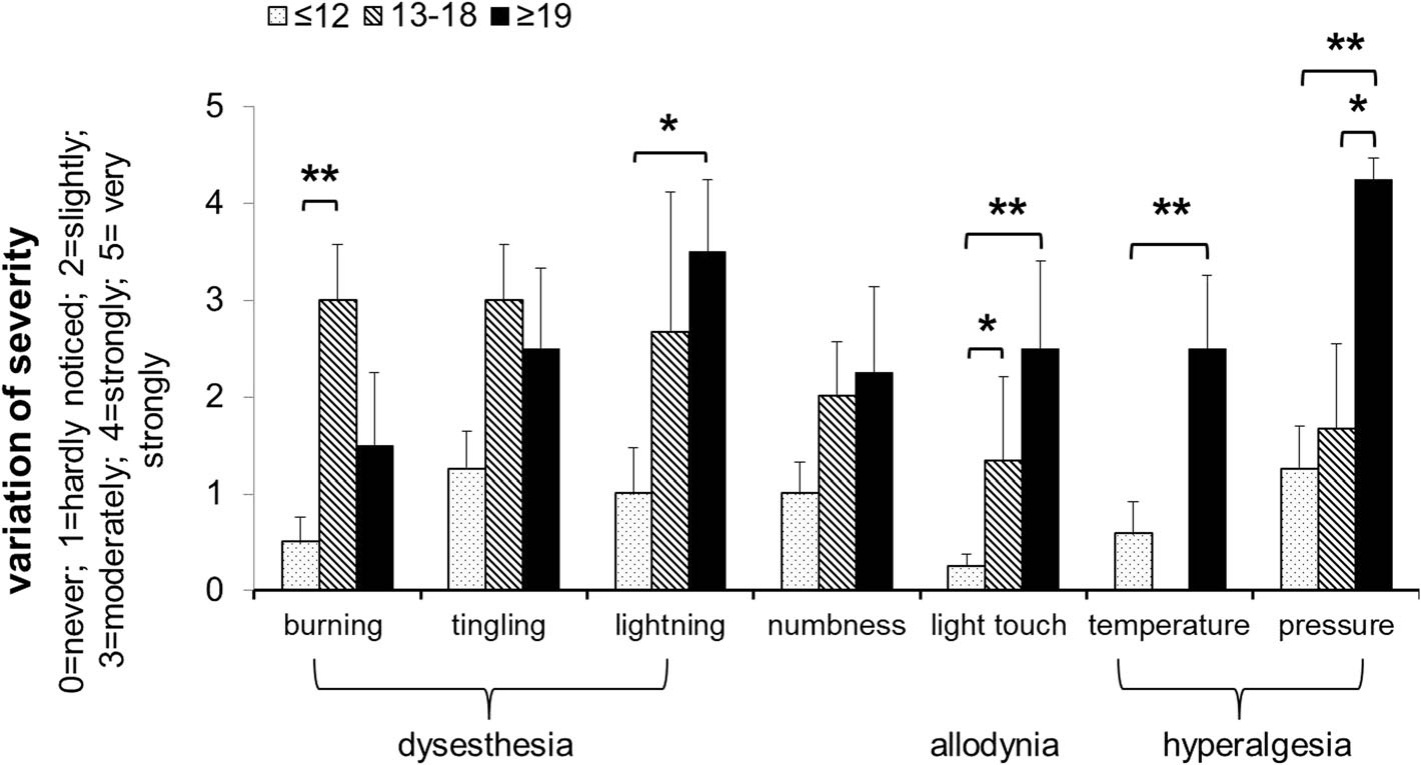
Variation of sensorial and pain perception in patients with myogenic TMD concerning their painDETECT score (≤ 12, *n* = 12; 13–18, *n* = 3; ≥ 19, *n* = 4); Variation of severity of sensational (dysaesthesia, numbness) and pain perception (allodynia, hyperalgesia) as revealed by the painDETECT questionnaire (1 = never, 2 = hardly noticed, 3 = slightly, 4 = moderately, 5 = strongly, 6 = very strongly); mean ± SD; Significance as results of analysis of variance (ANOVA) with LSD post hoc correction of multiple comparison (*p < 0.05; **p < 0.01; ***p < 0.001)

An analysis of the individual answers from the painDETECT questionnaire revealed similar variation in severity of pain perceptions for the distinct TMD groups (painDETECT ≤ 12; 13–18; ≥ 19). Particularly answers to questions concerning hyperalgesia, such as, “Is cold or heat in this area occasionally painful?”, “Does slight pressure in this area, e.g., with a finger, trigger pain?” [24] revealed that TMD patients with painDETECT scores ≥ 19 had moderately, strong or very strong enhancement of pain perception, whereas patients with painDETECT scores 13–18 and ≤ 12 answered these questions with “never”, “hardly noticed” or “slightly painful” (*p* < 0.05–0.01, Fig. 3).

### Pain history and current pain

All TMD patients with painDETECT scores ≥ 19 reported additional pain in other body regions already lasting for years (Table 2). On a numeric rating scale (NRS 0–10), the group with painDETECT scores ≥ 19 quoted the lowest present pain (1.8 ± 0.9) and the lowest pain maximum during the last week (1.8 ± 0.7) among all TMD groups. In contrast, the painDETECT 13–18 group with pain lasting for only 6–12 months showed the highest pain estimation for present pain (3.7 ± 1.2) and maximal pain of the last week (6.7 ± 1.1). In addition, this group showed the highest score for days per year when they were unable to work (> 50) (Table 2).

**Table 2:** Pain estimation of TMD patients (TMD all and according to their painDETECT scores (≤ 12, *n* = 12; 13–18, *n* = 3; ≥ 19, *n* = 4)); pain estimation in mean value ± SD or in mean % of 100%

Pain estimation (mean ± SD)	TMD	TMD - painDETECT		
	all (*n* = 19)	≤ 12 (*n* = 12)	13–18 (*n* = 3)	≥ 19 (*n* = 4)
present pain (NRS)	2.9 ± 0.5	3.2 ± 0.7	3.7 ± 1.2	1.8 ± 0.9
maximal pain - last week (NRS)	4.3 ± 0.6	4.6 ± 0.7	6.7 ± 1.1	1.8 ± 0.7
minimal pain - last week (NRS)	2.4 ± 0.4	2.7 ± 0.6	2.3 ± 0.7	1.5 ± 0.8
pain estimation BPQ 25 (1–13)/ sensory	8.1 ± 1.6	4.8 ± 1.1	18.3 ± 3.1	9.3 ± 2.9
pain estimation BPQ 25 (14–17)/ affective	3.5 ± 0.7	3.5 ± 1.0	3.7 ± 1.4	3.5 ± 1.3
pain estimation BPQ 25 (18–19)/ evaluative	2.7 ± 0.5	2.3 ± 0.6	4.3 ± 0.7	2.8 ± 1.2
pain estimation BPQ 25 (18 + 19 + 20/2)/ affective-evaluative	1.6 ± 0.3	1.4 ± 0.4	2.3 ± 0.3	1.5 ± 0.7
pain since	1–2 y	1–2 y	6–12 m	2–5 y
inability to work due to pain	12% (1–2 d)	45% (1d)	67% (> 50 d)	25% (1–5 d)
reduction of activity due to pain (work)	39%	36%	43%	48%
reduction of activity due to pain (leisure)	44%	46%	43%	40%
additonal pain in other body regions	67%	55%	67%	100%
pain-free time	min-h	min	h	d

*NRS* Numeric rating scale, *BPQ* Berne pain questionnaire (number 1–13 comprising sensory adjectives, maximum value 50; 14–17 comprising affective adjectives, maximum value 17; 18–19 comprising evaluative adjectives, maximum value 7; 20 comprising affective-evaluative adjectives, maximum value 5.5)

### Psychological comorbidity

The patients with a painDETECT score ≥ 19 showed the highest values for anxiety (mean 9.3 ± 7.0, *p* > 0.05, 50% > 10) and depression (mean 7.0 ± 3.2, *p* < 0.01) in the HADS-D among the TMD painDETECT groups (≤ 12, 13–18, ≥ 19) compared to healthy subjects (Table 3). Significant differences were found for the painDETECT ≥ 19 group for praying and hoping (CSQ5) (mean 4.1 ± 0.9, *p* < 0.05). The painDETECT group ≤ 12 showed significant values for catastrophizing (CSQ6) (mean 3.6 ± 2.1, *p* < 0.05) and the painDETECT group 13–18 showed significant values for increased behaviour activities (CSQ7) (5.4 ± 0.8, *p* < 0.05) (Table 3).

**Table 3:** Psychological Comorbidity of TMD patients (TMD all and according to their painDETECT scores (≤ 12, *n* = 12; 13–18, *n* = 3; ≥ 19, *n* = 4))

Psychological comorbidity		Healthy subjects	TMD		TMD - painDETECT					
		(*n* = 38)	all (*n* = 19)	*p*-value	≤ 12 (*n* = 12)	*p*-value	13–18 (*n* = 3)	*p*-value	≥ 19 (*n* = 4)	*p*-value
anxiety (HADS-D)	mean ± SD	5.0 ± 3	7.3 ± 5	0.082	7.1 ± 4.5	0.102	5.3 ± 3.1	0.865	9.3 ± 7.0	0.034*
	< 8	*n* = 32	*n* = 12		*n* = 8		*n* = 2		*n* = 2	
	8–10	*n* = 4	*n* = 0		*n* = 0		*n* = 0		*n* = 0	
	> 10	*n* = 2	*n* = 6		*n* = 3		*n* = 1		*n* = 2	
depression (HADS-D)	mean ± SD	2.2 ± 2.5	6.4 ± 4.3	0.001**	6.6 ± 5.1	0.000***	5.3 ± 3.1	0.105	7.0 ± 3.2	0.006**
	< 8	*n* = 37	*n* = 10		*n* = 6		*n* = 2		*n* = 2	
	8–10	*n* = 1	*n* = 5		*n* = 2		*n* = 1		*n* = 2	
	> 10	*n* = 0	*n* = 3		*n* = 3		*n* = 0		*n* = 0	
diverting attention (CSQ1)	mean ± SD	2.9 ± 1.1	3.3 ± 0.3	0.335	3.3 ± 1.4	0.423	3.4 ± 2	0.592	3.3 ± 1.6	0.634
reinterpreting pain sensation (CSQ2)	mean ± SD	2.2 ± 1	2.0 ± 0.2	0.584	2.2 ± 1.1	0.983	1.7 ± 1.1	0.499	1.7 ± 0.5	0.434
coping self-statement (CSQ3)	mean ± SD	3.7 ± 1.1	4.3 ± 0.4	0.157	4.2 ± 2	0.306	3.8 ± 0.5	0.932	4.9 ± 0.8	0.108
ignoring sensation (CSQ4)	mean ± SD	3.3 ± 1.2	3.3 ± 0.3	0.960	3.5 ± 1.8	0.783	2.3 ± 1.2	0.229	3.8 ± 1	0.497
praying and hoping (CSQ5)	mean ± SD	2.6 ± 1.3	3.0 ± 0.3	0.314	2.5 ± 1.4	0.872	3.1 ± 0.8	0.481	4.1 ± 0.9	0.030*
catastrophizing (CSQ6)	mean ± SD	2.0 ± 1.1	3.3 ± 0.4	0.022*	3.6 ± 2.1	0.006**	3.2 ± 2.1	0.193	2.6 ± 1.8	0.503
increased behaviour activities (CSQ7)	mean ± SD	3.5 ± 1.5	4.3 ± 0.3	0.080	4.1 ± 1.5	0.328	5.4 ± 0.8	0.044*	4.3 ± 1.6	0.320
pain behaviour (CSQ8)	mean ± SD	4.0 ± 1	3.7 ± 0.3	0.473	3.2 ± 1.2	0.055	5.1 ± 1.5	0.089	4.0 ± 0.6	0.967

*HADS-D* Hospital anxiety and depression scale, anxiety in mean value ± SD or divided in subgroups concerning their severity (< 8, 8–10, > 10), depression in mean value ± SD CSQ = Coping strategies in mean value ± SD; p-value for TMD all as results of unpaired t-test as related to healthy subjects considering the Levene’s test for equality of variances; *p*-value according to the painDETECT scores as results of analysis of variance (ANOVA) with LSD post hoc correction of multiple comparison (*p < 0.05; **p < 0.01; ***p < 0.001)

## Discussion

The present study disclosed pronounced somatosensory changes in a subgroup of TMD patients identified by their scores in the painDETECT questionnaire. Somatosensory changes in this subgroup with painDETECT scores ≥ 19 consisted of lowered thresholds for pressure pain as well as for cold and heat stimuli in the Quantitative Sensory Testing (QST). Additionally, in the painDETECT questionnaire, the questions concerning sensory gain (hyperalgesia) in particular were answered with high values. Similarly, Herpich et al. found that patients with more severe signs and symptoms of TMD had a lower pressure pain threshold [31]. Such somatosensory changes result in amplification of nociception, which promotes and sustains chronic pain states [18]. As we believe stress-induced sensitisation to be one reason for chronification of TMD [18, 32], we focused our attention not only on the signs of sensory gain (pressure, cold and heat hyperalgesia), but on psychological comorbidity of the TMD patients. We found that the patients with painDETECT scores ≥ 19 also showed the highest values for anxiety, depression, praying and hoping among all TMD patients.

In clinical examination we revealed no abnormalities in facial mimic expression for all TMD patients, but found pain sensitivity for mandibular movement and upon palpation of different muscles, the temporomandibular joint and the trigeminal foramina. Relating to clinical examination, we found a positive relationship between the increased sensitivity during masseter palpation and higher painDETECT scores. In the “painDETECT ≥ 19” group, the higher pain upon masseter palpation could be confirmed by measurement of pressure pain thresholds (PPT) in the Quantitative Sensory Testing (QST).

Our findings are supported by other studies in which several patients with chronic temporomandibular disorder were found to be more sensitive to painful stimuli than other patients or healthy subjects. Pfau et al., for example, distinguished a “sensitive” and an “insensitive” TMD subgroup with regard to their tenderness on palpation of orofacial muscles and trigeminal foramina and could confirm this hyperalgesia using QST [11]. Likewise, de Sequeira et al. assessed sensory characteristics of patients with chronic pain involving a combination of thermal, mechanical pain stimuli and found the majority of patients (73.3%) with pain upon craniofacial muscle palpation [33]. Several other research groups perceived the diagnostic value of pressure pain threshold analysis for TMD as well [14, 34–36]. QST is a method to investigate pressure pain thresholds and, therefore, is a useful component in mechanism-based classification of TMD. It is frequently mentioned for pain assessment and diagnosis in the orofacial region [36–40].

The fact that only the “painDETECT ≥ 19” group showed significantly decreased QST pressure pain thresholds (PPT), underlines the effective use of the painDETECT questionnaire in the differentiated analysis of TMD subgroups.

Until now the painDETECT questionnaire has not been validated for the trigeminal region. In accordance with our study, the painDETECT questionnaire was assessed for detecting neuropathic pain in patients with post-traumatic inferior alveolar nerve injury (IANI) and lingual nerve injury (LNI) [41]. The authors concluded the painDETECT questionnaire not to be suitable for screening IANI or LNI. Heo et al. 2015 found the painDETECT questionnaire, although not being an ideal principal screening tool for burning mouth syndrome (BMS), could still be useful to identify a substantial proportion of neuropathic symptoms in primary BMS patients [42].

In addition to the significantly increased PPT, a significant hyperalgesia against cold and heat painful stimuli could be revealed for patients with chronic TMD and painDETECT scores ≥ 19. Such an increased sensitivity against cold and heat pain, in addition to pressure hyperalgesia, was also found for the “sensitive” TMD pain group as distinguished by Pfau et al. 2009 [11], showing more than 11/18 tender points according to the former diagnostic criteria for FMS. As results from both the “sensitive” and the “painDETECT ≥ 19” TMD subgroup show that the pain is not only present in the area of the trigeminal nerve, but throughout the whole body, changes involving the central nervous system should be considered [43]. Central sensitisation might arise as a result of insufficient endogenous descending inhibition [18–20]. Persistent psychosocial stress and increased mental vulnerability, as found within the patients with painDETECT scores ≥ 19, may, therefore, be the cause [21, 32, 44]. This can explain the spreading of pain throughout the body and the increase during examination. The low subjective pain rating of these patients can be interpreted as adaption to the chronic pain state, although habituation to pain is not known. In addition, the “sensitive” and “painDETECT ≥ 19” group both show high levels of anxiety and depression, further evidence for central sensitisation. Therefore, we assume psychosocial factors to be one reason for chronification in both groups.

In our study, the painDETECT questionnaire enables the identification of subgroups of patients with chronic TMD showing different severity in induced pressure, cold and heat pain. Nevertheless, the neurobiological basis for this is not fully elucidated, but as we found psychological comorbidity for the patients, we assume stress-induced hyperalgesia to be the reason [45, 46]. Thermal hyperalgesia may be triggered by a stimulation of the dorsomedial hypothalamic nucleus, whereby the rostral ventromedial medulla plays an important role by activating so-called “ON-cells” that amplify the nociceptive input. This part of the brain is also responsible for allodynia and hyperalgesia in neuropathic pain [19]. Moreover, a number of other long-lasting changes in the neural system are probably involved in brain activity. The dysfunction of the hypothalamic-pituitary-adrenal axis (HPA axis) and various neurotransmitter systems in the brain, including the endogenous opioid system and the serotonergic and noradrenergic systems, have been demonstrated in animal experiments to be relevant factors [20]. Chronic TMD might be caused by hypersensitivity of the nervous system and central sensitization [47].

In our study we found all TMD patients showing signs of depression and anxiety. The scores were higher in TMD patients with increased painDETECT scores (≥ 19). In contrast to our findings, Reiter et al. analysed a less significant role of anxiety in patients with chronic TMD compared to depression and somatization [48]. Yu et al. 2015, however, detected high anxiety (OR 2.48; 95% CI 1.25–4.90) as the most significant factor associated with TMD. Among other publications indicating psychological stress to be significantly associated with TMD [49], Ismail et al. 2015 found clinical symptoms of depression in 16% of the TMD patients. They recommend the use of screening tools for psychological disorders on a regularly basis when evaluating TMD patients. Psychological stress is a risk factor for the development of a painful TMD [50]. The effects of stress on pain vary according to duration of exposure, mental and biological vulnerability of the individual and age at exposition. Effects are most pronounced in individuals whose genetic susceptibility increases responsiveness to catecholamine neurotransmitters [50]. Furthermore the large prospective OPPERA (Orofacial Pain: Prospective Evaluation and Risk Assessment) study found psychological variables to predict first onset of TMD [51]. Therefore, informing and educating patients about pain perception and functional jaw opening becomes essential in order to decrease fear and depression concerning TMD and to improve jaw function and quality of life [52].

## Conclusion

In our study we were able to prove the painDETECT questionnaire to be helpful as an additional diagnostic tool, which, together with QST, can reveal hyperalgesia for pressure (PPT) and thermal sensation (CPT, HPT) in chronic TMD patients. High painDETECT scores (≥ 19) correlate with decreased pain levels for pressure and thermal sensation in QST. As TMD patients of the painDETECT group ≥ 19 have additional pain in other parts of the body, suggesting central sensitization [53], we would recommend testing also an extra oral site when performing QST in these patients, for example the dorsum of the hand. An obvious limitation in the present study was the small number of patients, especially in the painDETECT 13–18 (*n* = 3) and ≥ 19 (*n* = 4) group.

## Abbreviations

ANOVA: Analysis of variance
BMS: Burning mouth syndrome
BPQ: Berne pain questionnaire
CDT: Cold detection threshold
CPT: Cold pain threshold
CSQ: Coping strategies questionnaire
DFNS: German Research Network on Neuropathic Pain
DMA: Dynamic mechanical allodynia
HADS-D: Hospital anxiety and depression scale
HPT: Heat pain threshold
IANI: Inferior alveolar nerve damage
LNI: Lingual nerve impairment
MDT: Mechanical detection threshold
MPS: Mechanical pain sensitivity
MPT: Mechanical pain threshold
PHS: Paradoxical heat sensation
PPT: Pressure pain threshold
QST: Quantitative Sensory Testing
SD: Standard deviation
SEM: Standard error of the mean
TMD: Temporomandibular disorders
TSL: Thermal sensory limen
VDT: Vibration detection threshold
WDT: Warm detection threshold
